# Challenging score-centered norms in Western classical higher music education: an exploratory qualitative study of instructor-led initiatives in the UK and Europe

**DOI:** 10.3389/fpsyg.2026.1768511

**Published:** 2026-03-10

**Authors:** Raluca Matei, Remi Chiu

**Affiliations:** 1 Birkbeck Business School, Birkbeck, University of London, London, United Kingdom; 2 Peabody Institute, Johns Hopkins University, Baltimore, MD, United States

**Keywords:** higher music education (HME), norm-challenging, score deviation, Western classical music (WCM), interpretive autonomy, improvisation, performance anxiety, conservatoire pedagogy

## Abstract

Western classical music (WCM) in higher music education (HME) remains anchored in score-centered norms that may constrain interpretative freedom. While innovative pedagogies exist, little is known about how instructors who challenge these norms design and sustain their courses, particularly within conservatoire settings. This exploratory qualitative study examines: (1) the characteristics and aims of such courses, (2) how they challenge WCM norms through their content, (3) instructors' perceptions of course impacts, and (4) the institutional contexts that enable or constrain these initiatives. Semi-structured interviews were conducted with 15 instructors across seven countries in Europe and the UK, predominantly from conservatoires, with three participants from university music departments. Analysis combined content analysis for course characteristics with inductive thematic analysis using a deductive-inductive framework for norm-challenging approaches, perceived impacts, and institutional contexts. Courses clustered into two broad categories: repertoire-based designs employing historically informed and ahistorical score deviation, and non-repertoire designs centered on improvisation and cross-arts practice. Instructors perceived positive impacts including what they described as reduced performance anxiety and increased student agency, alongside some student reticence toward excessive freedom. Institutional tensions were pervasive, including skepticism from colleagues and examination panels, the primacy of one-to-one instruction, funding pressures, and assessment regimes privileging accuracy. Adoption was facilitated by historical framing strategies, collegial alliances, leadership support, and flexible evaluation frameworks. Instructors expressed cautious optimism regarding long-term employability benefits while doubting immediate gains given prevailing audition and orchestral selection practices. It is important to note that findings represent the perspectives of a purposively sampled group of critically oriented instructors and should not be generalized to indicate prevalence or typicality across HME more broadly. The study contributes depth of understanding regarding how norm-challenging courses operate when they exist, rather than how common such courses are. Implications for curriculum design, assessment practices, and future research directions are discussed, including the need for direct investigation of student experiences and audience impacts.

## Introduction

Western classical music (WCM) faces significant challenges in maintaining relevance and reaching new audiences. Its future likely hinges on engaging younger, more socio-economically and racially diverse audiences and tackling what scholars have termed its “museum problem” of canon-bound historicism (Smith and Peters, 2024). Innovation in WCM performance remains cautious, constrained by canon priorities and score fidelity. While recent developments such as alternative venues, closer performer-audience interaction, and novel presentation and marketing strategies have made progress, classical concerts still tend to be impersonal, predictable, and biased toward established works (Pitts et al., 2024; Sloboda and Ford, 2018). Compared with theater's turn toward treating texts as springboards for experimentation, classical music generally lags in embracing similarly transformative performance practices (Gaunt et al., 2021).

Across Europe, the UK, and North America, critical debates on diversity, inclusion, gender equality, decolonizing the curriculum, and strengthening the student voice are already well underway [AEC, n.d.; AEC Student Working Group, 2017; Conservatoires UK, 2021; National Association of Schools of Music (NASM), n.d.]. Enduring issues of misogyny, classism, discrimination, and abuse compound these challenges. In higher music education (HME), the master-apprentice model concentrates power with studio teachers, who are often revered for their performance credentials despite limited pedagogical training (Norton et al., 2019). Meanwhile, musicians' mental health has been identified as a growing concern, with a 2023 European Union report noting especially high music-performance anxiety in classical settings, where flawless execution and fear of mistakes dominate, alongside risk factors such as limited social support, competition that suppresses individual artistic identity, low autonomy for orchestral players, and precarious employment conditions (European Union, 2023).

The current study investigates how some instructors of WCM perceive themselves to challenge the norms of the field, particularly with regard to score deviation, improvisation, and interpretive autonomy.

### Theoretical framework: norms in Western classical music

Our primary analytical lens draws on Leech-Wilkinson's (2020) systematic documentation of beliefs that classical musicians often internalize as unquestioned truths. These norms define WCM culture and frequently constrain performers' artistic freedom. Rooted in fidelity to scores and composers, and in the presumed superiority of historically informed performances, these norms include beliefs such as: everything necessary is contained in the score; the musical work transcends any individual performance; music is more authentic when performed in historically appropriate ways; the composer's knowledge is superior to that of performers; the composer's intentions are knowable and should guide interpretation; composers occupy an almost sacred status; and the performer's individual voice should remain subordinate to the work.

While Leech-Wilkinson's framework provides our primary analytical lens, we acknowledge that alternative perspectives exist for understanding how norms operate in music education contexts. Sociologists of music, such as Kingsbury (1988), emphasize the institutional reproduction of hierarchies and the cultural systems that sustain them. Curriculum theorists examine how particular forms of knowledge become legitimated or marginalized within conservatoires. Institutional studies highlight how organizational structures, funding mechanisms, and assessment regimes constrain pedagogical innovation (see Ford, 2010). Our choice of Leech-Wilkinson's framework reflects our particular interest in performer-focused, practice-embedded norms that directly shape how students learn to approach scores and performances, while recognizing that the institutional and sociological factors these other perspectives illuminate also shape the terrain our participants navigate.

### Performer wellbeing and pedagogical context

Research increasingly documents the psychological costs associated with WCM training, including performance anxiety, perfectionism, and identity threats that arise when artistic autonomy is constrained (European Union, 2023; Fujimoto and Uesaka, 2024, 2025). Reduced interpretive autonomy may undermine wellbeing, while existing literature suggests that interventions centring musical meaning, fostering belonging, and reframing audience relationships may reduce anxiety and support experiences of flow (Han et al., 2022; Hendricks et al., 2014; Ripani and Matei, in press^1^). These findings from prior research provide possible conceptual frameworks for understanding why some instructors may seek to develop pedagogies that expand interpretive autonomy, though the present study does not directly measure these psychological constructs.

The master-apprentice model that predominates in conservatoires presents particular challenges for student autonomy and wellbeing. One-to-one teaching often foregrounds teacher dominance, heavy technical focus, limited questioning, and restricted encouragement of independent learning (Burwell et al., 2019; Foletto, 2018; Gaunt, 2009). These dynamics may contribute to the internalization of externally imposed standards and the suppression of individual artistic voice. Courses that explicitly challenge these dynamics therefore warrant investigation not only for their pedagogical innovation but also for their potential contributions to student psychological health.

Cross-disciplinary work and collaborative pedagogy counter the master-apprentice norm and support broader, healthier musical identities (Rumiantsev et al., 2020; MacDonald and Saarikallio, 2024). Prior research suggests that improvisation may lower accuracy pressure, reduce fear of mistakes, and ease performance anxiety (Duffy, 2016). Brøske and Lysne (2019) document how collective improvisation in professional ensemble contexts can redistribute artistic ownership and challenge traditional hierarchies, offering models that may inform pedagogical approaches in higher education settings. These findings from improvisation research, ensemble studies, and collaborative learning likewise provide possible conceptual contexts for understanding why some instructors may seek to incorporate such approaches into their teaching, though, again, our study examines instructor perceptions of such approaches rather than measuring these psychological outcomes directly.

### Improvisation and historical practice

Improvisation occupies a central place in this study because it represents both a pedagogical tool and a site of ideological contestation within WCM. Historically, improvisation was integral to Western art music performance, from extemporized ornamentation to large-scale cadenzas. The current emphasis on precise text reproduction that avoids improvisation represents a historical anomaly rather than a timeless standard (Müller, 2023).

Reframing improvisation broadly—to encompass ornamentation, parameter variation in tempo, vibrato, articulation, dynamics, and timbre, continuo-style chordal realization, and thematic variation—positions it as legitimate creative practice rather than deviation from proper performance (Hill, 2017, 2018).

Other creative HME practices blur boundaries between listening, analysis, and performance; rethink performer–score relations; embed creativity within historically-informed performance (HIP); engage communities; use improvisation; and employ Dalcroze-style movement to support embodied interpretive choices (Burnard, 2014; Burnard and Haddon, 2016; Haddon and Burnard, 2016). Hutchinson and Howell (2016) similarly start analysis from students' experiences and metaphors, encouraging them to question assumptions and dissolve rigid splits between theory/analysis, performance, and composition.

Despite their value, many of these initiatives stop short of questioning the ideological foundations of canon interpretation. “Creative” performance remains bounded by tradition: in WCM, creativity is historically assigned to the composer, while performers adjust parameters (tempo, dynamics) rather than improvise or radically reimagine works (Ayerst, 2021; González-Moreno, 2013). Making WCM more inclusive therefore requires challenging aesthetic norms, especially score-fidelity, tightly coupled to deference to authority, reverence instead of pleasure, and distance from social concerns (Bull, 2024; Bull and Scharff, 2017, 2021). Students need a musical voice in interpretive decisions; otherwise, loyalty to deceased composers and their scores displaces creativity and may fuel performance anxiety (Leech-Wilkinson, 2016, 2020). Put simply, progress depends on scrutinizing the ideology of WCM, an issue flagged decades ago (Kivy, 1988; Young, 1988) yet still often taken for granted (Fujimoto and Uesaka, 2024).

### The present study

The purpose of this study was to explore whether courses exist in HME institutions that are specifically aimed at challenging the norms identified by Leech-Wilkinson (2020), and if so, to understand how they operate. We sought to answer the following research questions (RQ):

RQ1. What are the characteristics and aims of courses that challenge WCM norms (including location, institution type, level, optional or compulsory status, and when they were established)?
RQ2. How do these courses challenge norms through their content, even when instructors may not explicitly frame their work in these terms?
RQ3. What are the views of instructors regarding the impact of their courses on students?
RQ4. What are the views of instructors regarding the institutional contexts surrounding their courses, including factors that enable or constrain their work?

### Key terms and concepts

**HME** refers to a variety of institutions that include conservatoires, music colleges, music academies, music departments within broader universities, schools of music, and related nomenclature across different national contexts.

**Score deviation** refers to intentional departures from notated instructions in parameters such as tempo, dynamics, articulation, ornamentation, or structural elements. Such deviations range from historically informed practices, including Baroque ornamentation and Classical-era embellishment, to ahistorical reinterpretations involving radical manipulation of tempo, dynamics, or even structural reordering. Score deviation thus encompasses a spectrum from stylistically grounded historical practice to deliberately experimental approaches that question the authority of notation itself.

**Norm-challenging**, as operationalized in this study, was identified through two complementary processes. First, instructors self-identified as offering courses that challenge prevailing WCM norms through their response to recruitment materials. Second, researcher analysis applied Leech-Wilkinson's (2020) framework of documented WCM norms to examine course content and pedagogical approaches. This dual identification strategy means that what counts as norm-challenging reflects both practitioner self-perception and theoretically grounded analysis.

**Interpretive autonomy** refers to the degree of freedom performers exercise in making artistic decisions about how to realize a musical work. In WCM training, interpretive autonomy is often constrained by norms privileging fidelity to scores, adherence to teachers' interpretations, and conformity to established stylistic conventions (Leech-Wilkinson, 2020).

**Musicking**, following Small (1998), denotes the understanding that musical meaning emerges from the entire performance situation, encompassing social relationships, contexts, and modes of participation, rather than residing solely in the notated score or the sounds produced. This concept reframes performance as a socially embedded activity rather than merely the accurate reproduction of a composer's intentions.

## Materials and methods

### Design and rationale

We employed a qualitative, cross-sectional design using semi-structured interviews. This approach was appropriate given our exploratory aims: rather than testing hypotheses or establishing the prevalence of norm-challenging courses, we sought to map the landscape of instructor-led initiatives and understand how participants conceptualize and enact their practices. Qualitative methods are well-suited to capturing the complexity, context-dependence, and meaning-making processes involved in pedagogical innovation (Braun and Clarke, 2006, 2021). Fifteen interviews were conducted via Microsoft Teams, allowing participation from geographically dispersed instructors while providing the flexibility and rapport-building potential of synchronous video interaction.

### Participants and sampling

We used purposive sampling to recruit instructors of HME courses explicitly aiming to challenge WCM norms. This targeted approach was necessary given the specialist, possibly marginal, nature of such courses within conservatoire curricula. We recognize the inherent circularity in this design: participants were recruited because they self-identified as challenging norms, recruitment materials referenced examples aligned with Leech-Wilkinson's (2020) framework such as improvisation within repertoire and questioning score, composer, and audience relations, and our analysis subsequently applied this same framework deductively. This circularity does not invalidate the study, but it does circumscribe what the findings can claim: how a particular group of instructors conceptualize and enact norm-challenging practices, rather than demonstrating the prevalence or typicality of such practices across HME more broadly.

Inclusion criteria were as follows: participants must be currently teaching or have recently taught a tertiary-level course in HME; their course must explicitly address improvisation, score deviation, or critical examination of WCM performance norms; and they must be willing to be interviewed in English. Courses focused exclusively on jazz, popular music, or non-Western musical traditions were excluded, as were courses without structured pedagogical aims such as informal workshops.

Recruitment ran via social media, the first author's professional networks, institutional contacts, targeted emails, and snowballing. Prospective participants received an information sheet containing examples drawn from Leech-Wilkinson (2020), such as improvisation within repertoire and questioning relationships between score, composer, and audience. Participants could ask questions and then provided informed consent. The final sample comprised 15 instructors; this sample size is consistent with recommendations for thematic analysis seeking rich, contextualized accounts rather than statistical generalizability (Braun and Clarke, 2021). Participants worked across seven countries in Europe and the UK, predominantly from conservatoires, with three participants from university music departments. Twelve participants were male and three were female. To protect anonymity, locations are reported only as Europe or UK (see Table 1). Some terminology used by participants has been paraphrased where necessary to protect anonymity while preserving meaning.

**Table 1:** Characteristics of participants and their courses.

**Participant no**	**HME type**	**Location**	**Level (UG/PG) and other info**	**Optional/compulsory**	**When it started**	**Sex (M/F)**	**Interview length (min)**
P1	University Music Department	UK	UG (all), one semester, no genre basis	Optional	2011, but 2019 in its latest form	M	72
P2	University Music Department	UK	UG, final year, for advanced performance abilities, all instruments and singers	Optional	2016 (one year only)	M	60
P3	Conservatoire	Europe	UG & PG, for classical instrumentalists and singers (2 courses)	Optional	2001 and 2011, respectively	F	80
P4	Conservatoire	Europe	UG & PG, instrumentalists and singers, classical musicians	Optional	2006	M	66
P5	Conservatoire	UK	UG & PG	Different types (e.g., optional in year 1, compulsory in year 2)	1990s	M	62
P6	Conservatoire	Europe	PG, entire Master's Degree, all instruments	N/A	2016	M	60
P7	Conservatoire	UK	UG, composers and performers	Compulsory in years 1 and 2, optional afterwards	2020	M	52
P8	Conservatoire	UK	Mostly UG, mostly classical, some jazz musicians and dancers	Compulsory	2012	M	82
P9	Conservatoire	Europe	PG, classically trained doctoral students, instrumentalists and singers	Optional	2018 (one year long)	M	65
P10	Conservatoire	UK	UG (third and fourth years only), all classically trained instrumentalists	Optional	2019	F	65
P11	Conservatoire	Europe	PG	Entire Master's	2020	M	75
P12	Conservatoire	Europe	PG	Entire Master's (joint network of HME institutions across Europe and the US)	2011	M	45
P13	Conservatoire	UK	UG & PG	Optional at UG; entire Master's	2005	M	57
P14	Conservatoire	Europe	UG, first year, string players	Compulsory	2017	M	67
P15	University Music Department	UK	UG & PG	Optional	2014 (only until 2016)	F	66

### Procedure and interview guide

Semi-structured online interviews were conducted in English by the first author and covered: (1) course characteristics (e.g., aims, content, format, and student demographics); (2) pedagogical approaches to challenging norms; (3) perceived impacts on students; (4) and institutional context including enablers and barriers. Example questions included: “Tell us about your course (e.g., aims, who delivers it, why it started, why it is needed, when it started, how it is different from more standard courses)”; “Are there any assumptions or ideas that you are trying to challenge? If yes, what are these?”; “What is your general experience of the course (e.g., general evaluation, whether any changes were noticed as a result of the course, challenges encountered, what worked well)?”; and “What institutional support or resistance have you encountered?”. Probes encouraged participants to provide specific examples and to reflect on underlying assumptions.

Interviews were 63 min on average (range: 45–82; see Table 1). Interviews were transcribed using GoTranscript, accuracy-checked by the first author and the participants, anonymised, and assigned unique identification numbers.

### Data analysis

For RQ1, concerning course characteristics, we conducted content analysis following Hsieh and Shannon (2005), categorizing course characteristics, aims, formats, and contexts. For RQ2, concerning how courses challenge norms, we began with Leech-Wilkinson's (2020) framework of documented WCM norms, developing a complementary inductive framework as patterns emerged. For RQ3, concerning perceived impact, and RQ4, concerning institutional context, we applied inductive thematic analysis following Braun and Clarke (2006).

To enhance trustworthiness following Lincoln and Guba (1985), we employed several strategies. For credibility and transferability, we used purposive sampling to recruit participants. To enhance dependability, we maintained a transparent audit trail documenting analytical decisions throughout the process. Confirmability was supported through the inclusion of illustrative quotations and detailed descriptions of analytical procedures. Participants were invited to review their transcripts for accuracy.

We adopted a constructionist epistemological position, treating coding as interpretive rather than extractive (Braun and Clarke, 2019). Both authors coded collaboratively, using disciplinary differences (psychology; musicology) as analytic resources. Thematic analysis was undertaken thus: (1) Both authors familiarized themselves with all transcripts and discussed broad themes related to the research topic. (2) Transcripts were coded independently by both authors, with the first author coding exhaustively and the second author focusing specifically on musicological content. (3) Codes were discussed collaboratively, and the first author then inductively organized codes into themes aligned with our research questions. (4) The authors ensured that all relevant data segments had been appropriately coded and iteratively refined the themes until agreement was reached. Where new data did not align with the existing structure, the thematic framework was revised.

### Positionality

Both authors are trained musicians with complementary expertise (psychology/performing-arts health; musicology/historical performance) and share a critical stance toward WCM norms. The first author writes as a former performer with lived experience of constraints on autonomy; the second author as a historian attentive to the historical contingency of current practices. Both teach at HME institutions and prioritize critical thinking in their pedagogical work. This collaboration bridges humanities and sciences, linking practice, history, and psychology, and aligns with the study's multidisciplinary aims (see Parncutt, 2007).

### Ethical considerations

Ethical approval was obtained from the School of Business, Economics and Informatics Ethics Committee of Birkbeck, University of London, UK (ethics code: OPEA-20/21-18). All participants provided written informed consent prior to enrolment in the study. Given the sensitivity of institutional discussions, we prioritized anonymity. Two participants required anonymity to disclose their strategies.

## Results

This section presents findings organized by research question. Illustrative quotations are included with participant identification numbers. Interpretation follows in Discussion.

### RQ1: course characteristics and aims

Courses varied structurally (see Table 1). Established between 1990s and 2019, they were offered at both undergraduate and postgraduate levels. Most were optional electives, though some were compulsory. Two programmes constituted full master's degrees focused on innovative practice. Most courses were ongoing at the time of interviews, with a few having been time-limited.

Content analysis of stated course aims (Table 2) revealed four themes (Table 2). First, instructors cultivated performer freedom through improvisation and historical styles to widen interpretive options. Second, courses encouraged risk-taking and critical challenge to norms, including flexible score treatment. Third, instructors aimed to deepen connection to repertoire, claiming such goals as fostering experiences of flow, enjoyment, reducing performance anxiety, and enhancing performance quality. Fourth, courses aimed to broaden career pathways by developing transferable skills.

**Table 2:** Courses and how they challenge norms.

**Course no**	**Content**	**Aims**	**Reasons for inclusion based on (Leech-Wilkinson 2020)**	**Not challenging norms in any clear/explicit way**	**Does the instructor see themself as challenging norms? (Y/N)**
1	A performance class and seminar exploring music philosophy, interpretation, and improvisation, with some work on stagecraft and performance anxiety; students also choose either a short composition portfolio or chamber music (any genre); improvisation using modal scales and in Baroque styles.	To make students more independent from their teachers, more autodidact.	Treating the score as flexible: composite programming, interpretive pluralism, and viewing notation as inherently indeterminate (challenging fixity and boundaries). Questioning whether composers necessarily knew the “best” way their works should be performed. Challenging the idea of the musical “work” as an object; framing musical meaning as emerging from the whole environment, not only the sounds produced (akin to musicking). Positioning improvisation as both a mindset and a tool for student agency, supporting control, easing pressure, and reducing performance anxiety.	N/A	Yes
2	Students were introduced to historical models of alternative score performance and developed their own examples for a lecture-recital assessment. The course followed a structured, multi-level approach exploring concepts and techniques, supported by readings and listening, building skills, and guiding students toward their final presentations. Each session had two core parts: (1) practical examples tied to the students' repertoire, and (2) discussion of underlying principles, followed by hands-on work. In the first weeks, students completed short tasks such as listening to specific performers and imitating elements of their style. Other exercises included experimenting with tempo and loudness (including treating dynamics as motion), reflecting on what music is, and analyzing the language used to criticize performers, surfacing prejudices and unsupported claims. Students also used metaphor and character description to generate alternative interpretations, and shared experiences of being discouraged from following their own emotions and instincts in interpretation.	To design a course that gradually introduces students to the ideas behind rethinking score fidelity (e.g., why alternative approaches can be interesting, legitimate, and musically rewarding), and guides them step by step through practical techniques for exploring different ways of performing. Students will study the wide range of performance styles captured on recordings since the advent of sound recording and consider what this evidence implies for today's practice. They will be encouraged to question how historically grounded contemporary styles are, whether they truly transmit past traditions, and how closely they align with composers' expectations. The course aims to help students feel entitled to be creative with earlier repertoire by providing clear models of alternative approaches, ultimately fostering more imaginative interpretations and preparing them for careers where offering diverse performance options is an asset across genres and contexts beyond major institutions.	Assessment changes: exam regulations were amended to allow deliberately non-standard performances. Students had to notify examiners beforehand and briefly explain their approach. A full module was assessed via a non-standard performance. The core aim was student ownership of interpretation; success was defined as a convincing musical experience, whatever form it took. Style awareness: students imitated historical recordings to highlight past stylistic diversity, then explained how and why their own contemporary approach would differ. This fed into discussions of what “performance style” is and how it changes over time. Incremental experimentation: students made targeted, stepwise changes to one parameter at a time—radical tempo shifts, shaping and “motion” dynamics, rubato, and loudness—to test how different performances can still be musically coherent and embodied. Critiquing “musicality” norms: the course challenged the idea of musicality as a gatekeeping marker of conformity. Interpretive heuristics: students used metaphor and character description to generate alternative readings of a score and catalyze markedly different performances. Reframing success: ongoing discussion questioned assumptions that works can only “work” one way, that success equals correctness, that canonical scores have a single correct interpretation, and that fidelity to composers' intentions is either possible or inherently superior.	N/A	Yes
3	Creating bridges with multiple forms of art when teaching improvisation; asking students to musically represent poems, pictures; working on group dynamics and group work, musical group improvisation	To help musicians be freer in their playing, and less afraid of mistakes.	Challenging the idea and fear that you shouldn't be hitting “the wrong notes.” Maintain focus on metaphors, stories, characters, images and emotions (e.g., via improvisation) so that students do not get stuck on technique. Promoting/facilitating the idea of being in the flow, and focused whilst sensing what is happening (it's not analyzing). Asking students to improvise as warm up before a lesson. Then asking them to choose the parts of their improvisation that they liked the most and turn them into a composition—so, diminishing the gap between composers and performers. And challenging some of the students' assumptions that they were unable to compose.	Stays mostly away from performing repertoire.	Yes
4	Teaches improvisation incorporated into music theory/improve within classical music, e.g., improvised ornamentation in belcanto and Baroque music; asking students to play melodies based on harmonic structures/progressions and reduce existing pieces to their harmonic bases, aural training, musical analysis, asking students to compose.		Historically inspired improvisation (preferred over the vague term “classical improvisation”) within a music theory class: students improvise melodies over harmonic progressions, reduce existing works to their harmonic framework, develop aural skills and analysis, and compose, progressing to work with modes and non-diatonic scales. The improvisation focus is on musical meaning rather than right/wrong answers, narrowing the gap between performing and composing, training listeners to hear differently, and building stylistic fluency so performers can improvise convincingly within a score's language (e.g., cadenzas, preludes). It also supports historically grounded improvisation for idiosyncratic instruments with limited repertoire, foregrounding variation over a single “ideal” interpretation and normalizing the expectation of difference each time, while staying within style.	One of the aims is for students to master the score (and NOT to depart from it). Stays mostly away from performing repertoire and is always historically-informed.	Yes
5	Improvisation across the “languages” of multiple styles, Baroque, Classical, varied Romantic and post-Romantic idioms, modal, post-tonal, and tonally free, both solo and in groups. Students extemporize in different forms to understand styles “from the inside,” alongside theory. They create multiple structural/harmonic/motivic reductions of repertoire pieces, perform these reductions, and then improvise on them while developing their own interpretations.	To equip students with the stylistic “language” of different classical traditions so that they can improvise convincingly within a given style. This includes both expanding an existing score and creating original music that is grounded in a particular idiom. To deepen performers' connection with the music they play. To enhance the overall quality of performance by fostering greater stylistic awareness, responsiveness and expressive freedom.	Classical improvisation in performance. Deviations are always constrained by historical performance (how music was performed, expected to be, in the seventeenth, eighteenth, and most of the nineteenth century). Solo and group improvisation (independently of repertoire) within the languages of various styles (Baroque, classical, the different Romantic styles, post-Romantic, modal, post-tonal, tonally free).	Is always historically-informed.	Yes
	Work with actors is used to cultivate spontaneity in emotional expression, stage presence, and communication, and to connect musical elements with rhetorical gesture. This includes ensemble improvisation applied to standard repertoire, and exercises with singers acting the text in speech and calibrating expressive intensity to underlying harmony. A further strand explores non-verbal aspects of speech-like notation, phrasing, and meaning: musicians translate the “music” of actors' speech into tonally free sound.	To contribute to the revival of the lost art of classical improvisation and to enrich creativity in performance by combining practical know-how (structural, stylistic, textural and harmonic awareness) with real-time musical decision-making and spontaneous invention. To increase enjoyment and satisfaction in music-making for both performers and listeners.	Applying freedom in timing, timbre, taking decisions which are spontaneous, which are not reproducing the meticulous process of practice or preparing every micro beat in advance, but responds spontaneously to something that was done in the phrase before (active listening is a big part of it); then applying it to repertoire in duos for instance: one plays a Bach piece as it is, the other one applies the improvisation approach looking for the harmonic reduction, structure reduction, performing it. Improvisation focuses on active listening, the awareness of give-and-take in real-time. Aim: to enhance musicians' connectedness with what they play by facilitating the state of flow, thereby enhancing the quality of the performance. And to better synchronize with the audience. And for both performers and audiences to enjoy music more! Performers as creators, not executors of notes. Music as performance, not score/text. Challenging the focus on technique at the expense of expressive performance.		
6	Creativity in performance practice through discussion, guided listening, and practical experiments using conducting and piano rolls. Students bring their own repertoire for a masterclass-style format while engaging with philosophical questions about the score, the musical work, and the composer. The course also compares early recordings and piano rolls with modern performances, and uses conducting exercises that imitate interpretations that intentionally depart from the score.	Provide students with space to experiment and consciously develop their creative agency in performance. Using repertoire they are already preparing, students explore radical interpretive possibilities inspired by early recordings and linked to written accounts of nineteenth- and early twentieth -century performance practice. The focus is primarily nineteenth- and early twentieth -century repertoire, with occasional contemporary works where freer interpretation and stylistic re-imagining are especially pertinent.	The course draws on early recordings of nineteenth- and early twentieth-century repertoire (including piano rolls) and compares them with modern performances, using discussion to trace how performance practices have shifted over time. Students analyze changes in tempo, freedom, rhythmic flexibility, ornamentation, and portamento, sometimes using composer-led performances to show how composers themselves deviated from their scores. Practical activities include re-enacting specific historical interpretations (one student conducts while another plays) and, for composers like Brahms, listening to recordings of his students and discussing their stylistic choices. The goal is to support experimentation and develop students' creative agency in a safe, non-judgmental, collaborative setting, where they can try approaches often discouraged by one-to-one teachers or exam juries. Exercises include using tempo to shape character (guided by metaphor, story, or imagery), exploring ensemble freedom (“playing together without trying to	N/A	Yes
			play together”), and, at times, deliberately setting the score aside to prioritize the meaning students want to communicate, placing the performer at the center and treating the score as a resource rather than “the music.”		
7	Musicianship and improvisation that blur the boundaries between stylistic study, improvisation, and music theory: analysis, figured-bass writing, composing short sections/pieces in specific styles, and improvising within those stylistic frameworks.	To equip students with tools for interpretation and a clear sense that musical interpretation is not fixed, while highlighting improvisation as an integral part of musical practice.	Improvisation is integral to music-making, and performance styles evolve over time. Students therefore develop historically informed improvisation skills within specific idioms, including figured-bass realization. They learn Baroque and Classical techniques such as writing and realizing a figured bass, harmonizing a hymn/chorale in Baroque style, and composing a short Classical keyboard piece (e.g., a minuet).	Stays mostly away from performing repertoire and is always historically-informed.	No (but Yes from the perspective of the institution, in their view).
8	Each year, the institution pauses all one-to-one teaching, scheduled classes, and performance activity so the whole community can take part in a project-based course. Students and staff propose and run projects that challenge norms in classical performance (as well as dance and jazz), and participants choose which projects to join so students effectively lead the institution on a collective creative journey. Projects explore reworking “what's on the page” through practices such as musical collage, stretching time, using fragments of music with dance, and memorizing repertoire to transform it through movement. Other components include: a module in generative music (improvisation); seminars on music in society, authenticity, introductory ethnomusicology, and the roles music can play; a creative leadership strand focused on non-notated practice (e.g., conducted improvisation), using drama, games, and play to develop a shared improvisation vocabulary; Soundpainting and conduction (Butch Morris-style structural improvisation); a broad improvisation palette (Nordic modal, drone-based, Fluxus/Christian Wolff, maqam), plus critical reflection, cross-cultural listening, rhythmic training (models, unison work), and leadership practice in guiding groups.	To facilitate projects that challenge norms in classical performance and introduce students to a wider range of professional pathways they may not yet have considered.	Metaphors leading the music (question and answer; tension and release). Encouraging people to bring rhythms and sounds from different cultures. Challenging the idea that classical music is superior to other genres. Stays away from norms altogether, but also gives absolute freedom regarding repertoire/written music.	Stays away from repertoire.	Yes
9	Analysis of early twentieth-century performance styles through historically informed performance focused on late nineteenth–early twentieth century repertoire (where recordings exist), rather than the more typical HIP periods (Renaissance–Classical). The course draws on Leech-Wilkinson (The Changing Sound of Music), Neal	To give students an immersive sense of another era by inviting them to step into the shoes of past performers, without simply copying them. Students are encouraged to embody a particular style as closely as possible to understand its expressive norms and nuances. Historically informed performance is approached as an	Historically informed performance that helps students uncover expressive tools—vibrato, portamento, and varied forms of tempo rubato—that are rarely specified in the score but are central to late nineteenth–early twentieth-century interpretation. The aim is to expand students' interpretive options by reintroducing these	Does not go beyond recordings and HIP assumptions with regards to performance and	Yes
	Peres da Costa (Off the Record), and Robert Philip's work to introduce core questions and literature in performance-practice research. Students then explore early recorded performer “circles” (e.g., Carl Reinecke, Grieg, and Liszt's pupils such as Eugen d'Albert), using computer-assisted analysis (Sonic Visualiser) to identify performance patterns. They listen widely within a chosen era, compare recordings, and discuss findings. Assessment: students produce a recording, typically violin–piano duo, accompanied by notes documenting their interpretive choices and process.	experiment in adopting the conventions of a composer's time, while emphasizing that this is one valuable option among many, not the only legitimate way to perform a piece.	practices, moving beyond the mainstream modernist style dominant since the 1950s. Students listen extensively to recordings from the period and then attempt to perform in those styles. It also highlights Leech-Wilkinson's point that HIP is rarely applied once we reach twentieth-century repertoire, despite recordings giving us our first direct evidence of how performances actually sounded.	the interpretation of scores	
10	A course on innovation and performance styles, delivered through a small number of lecture/seminar sessions built around core ideas, with guest contributions from practitioners. Sessions are largely free-form (discussion and student presentations). Assessment includes: (1) a blog post in which students analyze an “innovative” performer as a case study, and (2) a reflective performance journal documenting lessons/rehearsals and key artistic conversations. Students are encouraged to take positions on controversial issues. Key themes include musical aesthetics, risk-taking vs. playing it safe, norms and “forbidden” performance practices, and performer creativity. Readings and case studies include Leech-Wilkinson (challenging performance; “style change” in the changing sound of music), Luca Chiantore, and performers such as Gilles Apap, Yuja Wang, and Patricia Kopatchinskaja, alongside media pieces (e.g., “Is playing it safe bad for classical music?” and coverage of Kopatchinskaja's competition controversies).	To challenge norms in music performance.	Introduces students to convention-breaking performers (e.g., Yuja Wang, Patricia Kopatchinskaja) and explores musicking, how recording technologies have reinforced unrealistic standards of perfection, and how performance styles change over time. The course supports students in clarifying their own artistic values and explicitly challenges white, masculine, and class-based norms, arguing that “challenging norms” should be integrated with, rather than separated from, diversity discussions. It debates issues such as creativity and diversity in classical music. It also critiques “my teacher says”-ism, the canon-centric belief that only masterworks matter, and the composer-as-god mindset, positioning improvisation and purposeful departures from the score as central to musicianship.	Academic course based on discussions only, no performance.	Yes
11	Improvisation across styles (Baroque, Classical, Romantic, Impressionist/early twentieth-century, late twentieth-century, and new music), with ensemble work that also uses improvisation to engage with social issues. The course includes a pedagogy strand (learning how to teach) and cross-arts collaborations.	To train students as ambassadors of improvisation in the world of classical music.	A form of historically informed practice that challenges mainstream interpretations. Students are expected to be able to create simple musical phrases, to “speak” the musical language, not just recite repertoire, countering the assumption that musicians only need to reproduce others' works rather than create their own as self-expression. Improvisation is used to deepen understanding of composition (harmony, style, and composers' ideas), support students in creating their own stylistically grounded material, and strengthen social bonding.	Stays away from repertoire mostly and is historically informed.	Yes
			The focus is on solo and chamber, classically informed improvisation, largely distinct from fixed repertoire: students work through historical idioms (Baroque, Classical, Romantic, Schubert, Debussy, Bartók, early twentieth century, possibly the Second Viennese School) before moving into a more “freestyle” approach that touches contemporary classical practice. It is also framed as a way to open music-making to amateurs, reviving an “amateur musician” ethos.		
12	A course on innovative practices with a substantial project component in which students take ownership of a major project. It includes mandatory strands on performance and communication (building audience connection); leading and guiding (collaborative and creative practice—working with groups, creating music, and improvisation, including community-based music-making), and entrepreneurship (preparing all master's students for their project work, covering project delivery alongside business, financial, and marketing aspects of professional practice).	To prepare students for professional practice by expanding their creative, entrepreneurial, and collaborative skillsets, and supporting them to carve their own paths.	Challenges the canon by prioritizing making new music rather than only reproducing it, and challenges interpretation by promoting diverse readings instead of repeated norms. It centers on a major student-owned professional project, supported by strands on: audience communication and connection; collaborative and creative practice (creating music with others), improvisation, and community work; project delivery, business/finance, marketing, and professional context; optional socially engaged music-making (e.g., music in hospitals). It encourages risk-taking and full ownership of innovative projects, giving students space to experiment with different artistic and professional roles (beyond the soloist model), develop broader tools for entrepreneurial practice, and connect their work to societal needs.	Stays away from repertoire.	They don't give a direct answer, preferring to avoid categorizing music. However, since creating new music has always been central to their career, the implicit answer is yes.
13	Improvisation, discussion and debate, and making music together, using gentle questioning and dialogue to help students unpack their assumptions, with a focus on the primacy of the ear and our relationship to notation.	To challenge Western classical music norms.	Music-making is framed as creative play and improvisation, prioritizing the ear over notation. Music comes before technique.	Doesn't deal with repertoire and is not for performers.	Yes
14	Free or tonal improvisation using structural tools such as dialogue and contrast, motif work, and chordal/tonal frameworks. Students draw on pop-song patterns and historical devices like the eighteenth-century Rule of the Octave, alongside analysis of Bach's solo violin sonatas/partitas and cello suites. Given a pattern, they create their own Baroque-sounding violin/cello preludes. Work includes both solo and group improvisation in the styles of various composers, grounded in detailed analytical study of those composers.	To teach improvisation in ways that deepen students' understanding of the scores they interpret and help them feel genuinely free on their instrument. The aim is to show that musicians can create compelling, technically grounded music without a written score, an approach closely aligned with historically informed performance principles.	Group and solo improvisation, both freestyle and historically informed, embedded in repertoire study to understand music beyond notated dynamics, through harmonic structure. Students improvise in historical idioms and in the styles of different composers, and create improvised “compositions” based on set patterns.	They view improvisation mainly as a tool for understanding the written piece more deeply, staying within historically informed practice and maintaining that the composer	No
				and score should be followed. The “original” score is treated as having a kind of sacral authority, and they see it as arrogant to assume one could improvise or compose something “better” than Bach or Beethoven.	
15	A series of performance-lab workshops grounded in the instructor's research. Students complete an adventure-education–inspired “comfort zone” worksheet, rating activities by how uncomfortable they feel, and co-create (and sign) a contract to ensure safety and confidentiality. The ethos emphasizes playfulness and not taking oneself too seriously. Activities include movement improvisation, interpretive dance, and theater (outside students' main idioms), non-idiomatic and avant-garde free improvisation, and idiomatic tasks such as radically reinterpreting a canon piece (classical/jazz/traditional). Exercises also involve extracting and recombining motifs in new orders, practicing intentional mistakes, and composing melodies.	To help students build the courage to take creative risks in music-making by stepping outside their comfort zones through fun, challenging performance activities. The aim is to reduce inhibitions and fear of mistakes, and to strengthen stage presence.	Questioning the assumption that improvisation “belongs” only to jazz, and challenging who gets to define which creative practices fit which idioms, arguing that this authority is shaped by race, gender, and class discrimination, and ultimately asking who is allowed to be creative. The underlying claim is that creativity is universal and not dependent on “talent.” Improvisation is defined broadly as any music-making where decisions are made in the moment, within an idiom, outside it, or through avant-garde free improve that deliberately avoids familiar conventions. Activities include: writing short compositions and sharing them, often composed by singing or playing to foreground aural/oral creation rather than rule-based writing; creating composite pieces by physically cutting motifs from existing music and reassembling them; play- and game-based work using movement, theater tools, and vocal exercises (including for instrumentalists); practicing intentional mistakes to reduce judgment, challenge perfectionism, and prioritize expressive impact and enjoyment over flawless note execution. The course also reframes the functions of music through discussion—valuing music not only as concertized or monetized output but also as enjoyment, expression, and community practice—and treats norm-breaking as an embodied, sometimes uncomfortable process of expanding one's self-defined limits of creativity.	Stays largely away from performing repertoire.	Yes

Overall, the courses prioritized critical thinking about notation and what Taruskin (1995) terms “modernist” style. In Text and Act, Taruskin argues that so-called historical performance is in fact the avant-garde wing of modern performance, a modernist, geometrical style characterized by textual literalism, clarity, impersonality, and an anti-romantic aesthetic, rather than genuine historical revival. Courses also emphasized intrinsic musical enjoyment and employability, aligning with broader HME goals and student priorities for high skill, individuality, and preparation for a diverse professional market (Polifonia Accreditation Working Group, 2010; Munnelly, 2020).

### RQ2: course content and approaches to challenging norms

Based on Leech-Wilkinson's (2020) framework, we identified several interconnected ways in which courses challenged prevailing norms. Courses reevaluated the score's authority by questioning the idea that notation is sacred and must be followed exactly, instead promoting interpretive pluralism, composite programming drawing from multiple sources, or radical alterations to tempo, dynamics, and phrasing, and by emphasizing improvisation within pieces or historical styles, highlighting variance over fixity. Courses challenged the idea of correct performance by arguing that different stylistic choices can be equally valid and by assessing performances based on how convincing they are rather than adherence to a prescribed style. Courses positioned improvisation as a core practice, whether historically informed or as free exploration, viewing it as a pathway to developing musicianship and creativity and, according to their accounts, to reducing performance anxiety.

Courses also deconstructed traditional hierarchies and cultural biases, with some actively challenging white, male, and class-based norms in classical music by incorporating non-Western music, non-canonical repertoire, and discussion of how classical music has historically positioned itself as culturally superior. Finally, courses promoted performance as a social and interactive act, with many emphasizing ensemble playing and communication over individual technical perfection, and some incorporating experimental approaches such as playing together with flexible rhythm or engaging with audiences in non-traditional ways.

Beyond Leech-Wilkinson's framework, we developed a complementary inductive categorization based on how courses engaged with scores, which we took as a criterion central to WCM ideology. This yielded two broad categories: non-repertoire designs that center improvisation and cross-arts practice; and repertoire-based designs that work directly with scores. Several courses span both categories, reflecting their experimental combination of strategies.

**Non-repertoire designs** (P3, P4, P8, P11, P12, P13, P14, P15). These courses stayed away from performing existing repertoire, instead centring improvisation, cross-arts practice, and collaborative creation. Improvisation could be based on stylistic, melodic, or harmonic structures that come from the Western classical music tradition and that tend to be historically-informed (P3, P4, P7, P11, P14) or it could be broader and not expected to adhere to a performance style or genre within Western Classical Music. Instead, it could include making music together; going beyond music, into cross-arts/cross-genre interactions and collaborations with other artists; project-based; the consideration of music in light of societal issues (P3, P8, P11, P12, P13, P15).

One course (P3) used poems and images as prompts for improvisation, to help quiet the inner critic and foreground expression over technical concerns. The course rejected hard boundaries between performing, composing, conducting, improvising, and working with notation, instead stressing their shared ground.

Another course (P8) employed student-led, play-based approaches where learners proposed projects and built a shared improvisational language through various structured improvisation techniques, including conducted improvisation and collaborative games. Leadership rotated among students, with structured reflection built into each session. Though housed in a performance-focused institution, the course deliberately foregrounded process, collaboration, and diverse professional pathways rather than polished final products.

One course (P11) addressed social issues through group improvisation while developing students' teaching and facilitation skills and sparking cross-arts collaborations. The course was aimed at training students as advocates for improvisation in classical contexts, countering the prevailing focus on repertoire and technique while strengthening peer bonds and reopening space for informed amateur participation alongside professional training.

An interdisciplinary, concept-driven course (P12) brought together students from jazz, classical, sonology, and composition backgrounds to co-create risk-embracing projects. Projects included adapted performance settings, construction of DIY instruments, genre fusions combining art song repertoire with jazz interpretation or Baroque works with electric guitar, and real-world applications such as radio plays, concert management, and community projects. Project-based learning integrated audience communication, entrepreneurship including financing and marketing, and sustained mentorship.

**Repertoire-based designs** (P1, P2, P5, P6, P9, and P10). These courses engaged directly with existing scores and compositions while encouraging various forms of deviation from standard performance expectations. Some emphasized historically informed deviation (P2, P5, P6, P9), including a course based on twentieth-century performance styles only (P9) drawing on evidence of past performance practices to legitimate departures from literal score reproduction. Instructors in this category introduced students to historical ornamentation, Baroque improvisation techniques, Classical-era embellishment, and parameter variation grounded in documented historical styles. This was done via the exploration of piano rolls and the use of tools such as the Sonic Visualiser for tule-finding and stylistic inference. Historical evidence thus served as a rationale for departure from contemporary norms of strict score fidelity.

Other repertoire-based courses encouraged deviation beyond historical evidence (P1, P2, P6, and P10), inviting experiments in radical tempo manipulation, unconventional dynamic shaping, or deliberate alteration of musical parameters for expressive purposes. Course 2 (P2) explicitly rejected the historical mandate, inviting any persuasive manipulation of parameters including loudness and tempo. Assessment in some cases was modified to accommodate non-standard performances, with students notifying examiners beforehand and briefly explaining their approach. The core aim was student ownership of interpretation, with success defined as creating a convincing musical experience. Reflective discussion and group interaction was identified as key mechanisms for generating ideas, testing assumptions, and co-designing alternative practices.

### RQ3: instructors' perceptions of course impact

Here, we identified three themes: perceived positive impact and student growth; student reticence; and employability: the good and the bad.

#### Perceived positive impact and student growth

Participants reported strong student feedback and perceived usefulness of their courses. Some students valued finally addressing topics they “discuss in the hallways anyway” (P10), and students often returned to instructors after noticing positive changes, even when they had initially joined by accident or felt uneasy about improvisation. One instructor observed that students “find their musicianship” (P3) through the work, while another noted students beginning to “connect to their inner selves” (P5) and “start asking about the meaning of what we're doing” (P11).

Many instructors reported perceiving “a little bit more self-confidence, and a little bit less anxiety” (P15) among students, along with purported reduced performance anxiety and more intense, focused performances. One participant explained that, in their observation, as relaxation improved, musical attention deepened: “So the interpretation is more powerful that way too” (P3). In their accounts, instructors perceived that students shifted emphasis from technique to interpretation and audience connection, with greater artistic freedom appearing, from the instructors' perspective, to soften the inner critic and encourage risk-taking and group attunement: “So they dare to take risks… they listen 80% more… they accept their ideas more quickly… they are more free. And the performance is like 90% more intensive” (P3).

One instructor noted a perceived shift toward student agency, describing how a student “actually asked their studio teacher why” (P10) in response to an instruction. Instructors also described what they perceived as stronger score connection through hands-on structural analysis: “not the theoretical or philosophical analysis, but hands-on analysis in real-time” (P5). Another reported benefit was opening up new ways to interpret scores and recognize notation's indeterminacy, with courses providing students “a vocabulary for feelings they already had” (P10) and prompting shifts in performance style.

#### Student reticence

Some students were reported to be indifferent or resistant to course content. One instructor described students who “could not handle a certain liberty […] or had different expectations” (P12). A few students felt uncomfortable, though they were not forced to participate, and highly technique-focused students often avoided these courses. One instructor recounted a student who refused outright, saying “I'm playing the Dvorák Cello Concerto… Why should I improvise bad music?” (P14).

#### Employability: the good and the bad

Instructors perceived potential employability gains in widening students' professional options through cross-genre improvisation skills, adaptable pedagogy, and preparation for portfolio careers suited to a freelance-heavy market. They also recognized industry constraints, noting that auditions reward narrow norms, so students must balance developing independence with learning to fit in when necessary. One course (P12) produced concrete outcomes: alumni formed ensembles and theater groups, led community projects, produced radio plays, created a vocal group for long-COVID patients, arranged art songs in jazz settings, built electro-acoustic instruments, and paired Baroque repertoire with electric guitar. Some could even seek funding to apply these skills professionally.

However, some instructors doubted immediate employability gains, noting these courses can run “in the opposite direction of the standards” (P6). Students must succeed within a rigid system; orchestral auditions reward conformity, so improvisatory or radical approaches may have limited short-term payoff. As one instructor noted, “in an audition, I'm still going to play my Joachim cadenza, and the way my teacher taught me” (P10). Instructors sought to balance fostering independent thinking with reality, recognizing that students “exist within this system, and… try to succeed within it” (P10). Another instructor expressed cautious optimism: “in the long term… performance practice norms will be indeed challenged… There are already signs of it in the classical music industry” (P9).

### RQ4: instructors' perceptions of institutional context

We identified three themes concerning institutional context: tension between innovative courses and wider institutional culture; a manifesto in disguise: shielding innovation for change; and facilitating change through institutional forces.

#### Tension between the innovative courses and the institutional culture

Participants described a “battleground for competing ideologies” (P15) between conservatoire expectations and the experimental work in their classes. They cited resistance from juries and teachers to unconventional interpretation: a student who improvised in a Master's exam was suspected of using prewritten material; others avoided improvising cadenzas “because they think the teachers will disapprove” (P4) or feared, “if I play this way my teacher will kill me” (P14). After a recital combining movements from Gade and Grieg into a single “composite sonata,” one instructor worried the reaction would be, “can you really do that?” (P1).

Instructors noted that terms like collaboration or interdisciplinarity can trigger defensiveness from colleagues, who dismissed these courses as “too childish,” “not serious enough,” or “not really appropriate,” especially compared with instrumental lessons and orchestral sessions “that are always first” (P3, P9). Objections were often framed as “not my way of doing” (P3). More broadly, a conservative culture, sometimes reinforced by senior studio teachers, meant “even some of the young people… are so often conservative,” which the participant found to be “disappointing” (P9).

Some participants felt senior staff were less willing to question assumptions or “not be so literal” about notation, noting that “age and where these people are in their career has got a significant role to play” (P1). Others described traditional colleagues feeling threatened by innovation: “when we started this… there were some eyebrows raised… ‘How come you call yourself innovative? We are also innovative”' (P12). Some studio teachers' reservations were understandable: “What if students think we're encouraging inaccurate playing?” (P5).

Instructors noted that the experimental content of their courses can create tensions with what students encounter elsewhere in their training. They felt responsible for offering students an alternative perspective while recognizing that students must ultimately navigate a system that may not share these values. Tellingly, improvisation was often positioned at the institutional margins, useful but carefully contained so it would not interfere with core repertoire training. When funding is limited, conservatoires run optional creative modules only intermittently. One instructor noted their course runs “every second year” (P3) due to resource constraints.

#### A manifesto in disguise: shielding innovation for change

Wary of limited institutional acceptance, some instructors adopted strategies to protect their work, drawing motivation from positive changes over time. The appeal to historical sources was “a useful getaway and academic grounding” (P6) to satisfy gatekeepers while those very sources were used to question norms. Vague course descriptions were used for “hiding” actual course content (P6). These strategies aimed to pre-empt charges of not being “academic enough” (P6) within conservative systems. Students‘ positive feedback also protected courses from being sidelined: “my students' positive feedback is like shields for me… colleagues hear that the students like it very much and they don't dare say anything” (P9).

Innovations were most vulnerable when seen to threaten what was perceived as the core business of one-to-one repertoire and technique training. Participants described a pragmatic approach to institutional navigation: resist pressures to conform, persist over time, and build coalitions. They stressed personal resolve: “I'm pretty good at resisting pressure to conform” (P13); “the alternative to conforming is not to leave, but to subvert” (P10). Patient networking was essential: “lots of cups of coffee and talks” (P8) to find allies. Collegial endorsement proved pivotal, with one instructor noting “Without them, it's just me railing against the system” (P10). Insider credibility also helped; coming from within the system as a classically trained musician meant ideas were not dismissed as external impositions (P11).

Despite challenges, participants deliberately created space to discuss tensions, knowing change would be slow and fragile beyond their courses. One hoped the class “equip[ped] the students with the vocabulary, the critical stance, to be able to challenge the authority of the teacher” (P10). They were committed to persistence—“I would not quit… before I retire” (P3)—and to aiming for “more impact down the line” (P6). Small shifts were seen as worthwhile: “even… perform one fingering differently… in a performance… baby steps” (P10); “a very limited but… personal impact on some older students… makes it worthwhile” (P6); “My responsibility is to inform the students and it's up to them to take decisions when they play” (P14). In practice, they carved time for dialogue and trusted students “to separate the two things… improvisation… could, but doesn't have to interfere with their repertoire” (P7).

Finally, some participants described a clear shift from skepticism to support over time, which felt encouraging. What once sounded “crazy” two decades ago (P3) has become more widely accepted at some institutions: “I am… surrounded by world-class colleagues who are… very supportive” (P5). At some institutions, improvisation moved from the margins to become a recognized strand: “whoever can come and do a minor in improvisation… they take it… serious” (P3). The “big fight” (P3) felt worthwhile as colleagues observed tangible benefits, with students becoming “more free” and finding the work “useful,” which in turn opened more colleagues to these ideas.

#### Facilitating change through institutional forces

Implementation was eased in institutions that were unconventional, research-oriented, and openly interdisciplinary, with flexible assessment and leadership that valued innovation. Cultural fit mattered: some systems had a fresh openness to improvisation: “in the year 2000, there was a new spirit to encourage improvisation” (P4); curricula were changed so “teachers [can] teach these kinds of improvisation skills” (P3). Freedom also increased where the course was not core business or the institution lacked a traditional music degree, enabling greater autonomy (“I'm given free rein to do what I want,” P13) and support from research units that “promote experimentation” and provide safe spaces for trial without high-stakes performance pressure (P6). Participants described “reflective,” “forward-looking communities” (P13, P15) “progressing toward improvising” (P7), with some courses becoming part of the institutional brand: “External people… say ‘This is amazing”' (P8).

Assessment flexibility was identified as a key enabling factor. Institutions that assess “music-making” broadly, instead of siloing performers/composers/conductors, can mark diverse outcomes fairly: students “doing quite different things… are all getting marked fairly… in the field of making music” (P1). Some exam regulations explicitly supported non-standard performances and encouraged students to briefly flag their intent and approach to examiners (P2). Others allow individualized assessment routines and non-text submissions (P13). Coupled with a curriculum “playground” for piloting new courses (P12) and supportive management that protects successful offerings, even amid cuts, these structures make innovation stick (P7, P10).

## Discussion

This exploratory qualitative study examined how 15 instructors across Europe and the UK design and deliver courses that challenge score-centered norms in Western classical higher music education. We found diverse pedagogical innovations emerging within and against constraining institutional contexts. Here, we situate these findings in the wider literature, outline contributions and limitations, and propose directions for future research.

### RQ2: course content and approaches to challenging norms

Drawing on Leech-Wilkinson (2020), these courses challenged score authority, legitimized multiple readings, and promoted improvisation, HIP-informed stylistic flexibility, and experimental or socially engaged performance. We distinguish repertoire-based courses (historical and ahistorical deviation) from non-repertoire courses (improvisation and cross-arts work), and examine perceived impacts and enabling conditions. Because most participants taught in conservatoires (12/15), we focus on innovation in these often change-resistant settings.

We treat norm-challenging as a continuum, not a binary. As Leech-Wilkinson notes, motivic improvisation can sit close to prevailing norms, and harmonic improvisation may be ahistorical even when anchored to a score's harmony. One instructor framed their approach as traditional, since improvisation was once commonplace, underscoring that “challenge” is context-relative (institution, geography, sector, compulsory vs. elective). Notably, few participants both worked with canonical repertoire and pushed beyond historically justified deviation, suggesting such practices remain rare.

The categories we propose overlap in practice; course 11 (P11) combined historically-framed improvisation with free improve and cross-arts work, while course 2 (P2) mixed historical and ahistorical approaches. Cross-arts projects can question norms by decentring music, but they need not deviate from the score where music is involved. Even courses avoiding repertoire often expected improvisation to be historically informed, suggesting norms persist when historical authority remains central.

Among repertoire-based courses, we observed tools that widened interpretative space, from subtle extensions to blurred boundaries between performance/composition and various forms of improvisation, ranging from tightly rule-bound to open-ended. Recreating nineteenth/early-twentieth-century styles can both support HIP and expose the contingency of modern “correctness.” Notably, only course 2 (P2) explicitly rejected the historical mandate, inviting any persuasive manipulation of parameters (e.g., loudness, tempo). Across courses, reflective discussion and group interaction were key to generating ideas, testing assumptions, and co-designing alternative practices.

#### Courses that encouraged deviations from the score in a historically-informed manner

A performer's style emerges from instrument affordances, practiced bodily capacities, and artistic choice, all shaped by period taste (Leech-Wilkinson, 2009). Early discs and piano rolls reveal style's contingency, so comparative listening trains students to hear convention as mutable. Framed as inquiry, imitation treats recordings as evidence: copy phrasing/timing, then infer period “rules,” a process that can prompt self-questioning and greater agency (Lisboa et al., 2005; Leech-Wilkinson, 2009). Because recording also standardizes expectations, models must be plural and critically weighed, not definitive (Philip, 2004). Historical media (e.g., piano rolls; course 6) and computer-assisted analysis (Sonic Visualiser; course 9) help extract performance principles, read against their technologies, then either re-embody them as historically plausible options or use them to underscore stylistic temporariness (Cannam et al., 2010; Peres da Costa, 2012; Philip, 1992).

Course 5 (P5) treated historical styles as grammars, not scripts: students reduced pieces to harmonic scaffolds, then rebuilt them with ornaments/cadenzas using cadential schemata and thoroughbass, achieving freedom within constraints (Butt, 2002; Haynes, 2007). This raises authority questions: historical “ingredients” are a start (Philip, 2004), but performers can choose, and justify, their own authorities and audiences (Leech-Wilkinson, 2020). Course 6 (P6) combined masterclasses with seminars comparing early/modern recordings, debating score–composer–work, and imitating score-divergent readings to test norms: if the “work” is a script, multiple readings are expected and “authenticity” is no single mandate (Taruskin, 1995; Leech-Wilkinson, 2009; Walls, 2003).

Course 9 (P9) applied a research-led HIP to late-nineteenth/early-twentieth-century style (Reinecke, Grieg, d'Albert), treating early recordings and texts as co-evidence of radical creativity (rubato, asynchrony, portamento, flexible tone/timing) and deriving transferable principles for assessment and teaching (Leech-Wilkinson, 2009; Philip, 1992, 2004; Peres da Costa, 2012). Overall, HIP functions as an evidence-rich toolkit to cultivate plural, defensible interpretations, not a prescriptive yardstick, an ethos most explicit in courses 2 (P2) and 6 (P6), with courses 5 (P5) and 9 (P9) leaning more into promoting HIP (Butt, 2002; Taruskin, 1995).

#### Courses that encouraged deviations beyond historically-informed performance (HIP) choices

Borrowing is foundational across eras/genres (Burkholder, 2001); “recycling” existing ideas is a core creative engine (Hill, 2018). If performance is creative practice (Cook, 2013) and musicking is socially situated (Small, 1998), performers are free to reshape works. Course 2 echoes critiques of text-centrism and its gatekeeping (including sexist/racist tropes) that constrain timing, dynamics, and articulation, parameters vital to shaping emotion and audience response (Leech-Wilkinson, 2020; Cook, 2013; Juslin and Laukka, 2003). Course 6 (P6) directly challenged the narrow interpretive space sustained by authenticity ideologies (Cook, 2013; Taruskin, 1995). Course 10 (P10) aligns with critiques of the work-concept, reframing performance as social action rather than fixed product (Goehr, 1994; Leech-Wilkinson, 2020; Weber, 2008; Small, 1998).

#### Non-repertoire designs

Courses 3 (P3) and 4 (P4) used harmony-first scaffolds (cadences, outlines, partimento) to make structure audible and generate material, treating harmonic “skeletons” as prompts for melodic improvisation (Gjerdingen, 2007; Sanguinetti, 2012). Course 4 (P4) also narrowed the performer–composer gap: students learned a style's idiom to improvise cadenzas, preludes, and inserts within it. Course 7 (P7) explicitly paired aural training, analysis, and composition with hands-on improvisation, assuming improvisation is integral and styles evolve. Course 11 (P11) aimed for musical fluency: speaking the language, not just reciting works.

Course 14 (P14) used rule-based patterning as historically grounded constraints. The rule of the octave links scale degrees to characteristic harmonies, letting students harmonize any diatonic line (Christensen, 1992). The method was as follows: analyze to set constraints, reduce for clarity, use schemata as generative units, then improvise for fluent realization (Sanguinetti, 2012; Gjerdingen, 2007). This bridges theory and improvisatory practice (Sánchez-Kisielewska, 2017). Here, improvisation deepens understanding of the written work, with the composer centered, and the instructor does not view this as norm-challenging.

Course 3 (P3), aligning with Small's musicking, treats meaning as socially situated rather than “in the notes.” Non-musical prompts (poems/images) were described by the instructor as quieting the inner critic and foregrounding expression, an approach that resonates with Leech-Wilkinson's invitation to learn from acting, experimenting with character, mood, even deliberate rule-bending (e.g., questioning a single Baroque Affekt or allowing “a little vulgarity”; Leech-Wilkinson, 2020, p. 204). For instance, Dalcroze Eurythmics builds embodied understanding/memory (Ridout and Habron, 2020) and has been linked in prior research to stronger ensemble rapport, listening, sound, and wellbeing (Wentink and Van der Merwe, 2020).

Improvisation's sociality is central: ensembles co-create via shared intentions and continuous mutual responsiveness; at peaks, attention distributes and the group “plays as one” (Lewis and Piekut, 2016). Limited chances for collective decision-making can leave some students unheard (Bull, 2024), while “student-as-creator” can overwhelm those needing clearer mentorship (Ski-Berg et al., 2024). Improvising outside the canon (course 11) may still press WCM by inhabiting its styles while sidestepping repertoire, but score-deviating approaches (course 15) confront core norms more directly. Learner-voice research highlights choice, experiential work, and teaching others (Després and Dubé, 2020), and Bull (2024) argues for more freedom in both repertoire and interpretation.

Performance as craft positions creativity in process: performers work with materials (notation, bodies, spaces) through iterative, worldly engagement rather than mere execution (Payne, 2016, 2017). Blended cohorts and concept-first projects (course 12) normalize performer-devised practice, link improvisation to real-world pathways, and de-silo genres (Lewis and Piekut, 2016). Pedagogical models for “reworking the canon” (Palmer, 2016; Sangiorgio, 2023) demonstrate how structural analysis can inform improvisation and collaborative (re)composition. Palmer notes that such an approach can sit uneasily with accountability cultures that demand clear, easily measurable outputs.

Finally, reframing the conservatoire as social and collaborative (Gaunt and Westerlund, 2013; Gaunt et al., 2021) shifts learning from the one-to-one “gold standard” toward distributed expertise across peers, ensembles, staff, spaces, and assessment. Aural skills can include communication and collaboration (Lebler, 2013); peer-studio and teacher-facilitated environments open space for experimentation and mutual support (Collens and Creech, 2013; Aho, 2013), and align with course 8's peer-led model.

### RQ3: instructors' perceptions of course impact

#### Positive impact and student growth

Instructors' accounts of positive impacts may be interpreted alongside existing research linking interpretive autonomy to performer wellbeing. Prior literature suggests that psychologically safe, inclusive climates support risk-taking, creativity, intrinsic motivation, and participation; authoritarian and competitive settings can suppress creativity and enjoyment, and reduced autonomy may undermine wellbeing (Han et al., 2022; Hendricks et al., 2014; Fujimoto and Uesaka, 2024, 2025; Leech-Wilkinson, 2020). This body of work provides a theoretical framework that may help contextualize the experiences instructors reported, though our data do not directly measure these constructs.

Framing musicianship as communication, not just flawless technique, echoes Small's musicking concept, where meaning arises from the whole performance situation. Technical precision can be decoupled from creativity, challenging conservatoire norms that privilege rule-following over individuality (Fujimoto and Uesaka, 2025; Leech-Wilkinson, 2020). External focus (audience/expressivity) may better enhance expressiveness and execution, than an internal, technique-focused mindset, though findings in this research area are mixed (Mornell and Wulf, 2018; Hohagen and Immerz, 2024; Lubert et al., 2024).

Instructors' descriptions of increased listening and risk-taking among students show conceptual parallels with what researchers have termed an “improvisatory state of mind”: classically improvised performances of Schubert (flexible timing/dynamics) produced more automatic control, enjoyment, and lower anxiety, and were judged to be more convincing in one experimental study (Dolan et al., 2018). These experiences that instructors reported share thematic similarities with documented flow correlates—concentration, clear goals, self-efficacy, openness, supportive climates, and skills–challenge balance—while comparison, value-disconnection, rigidity, peer conflict, and worry have been shown to inhibit flow (Ripani and Matei, in press) (see text footnote 1). Existing research suggests that interventions centring musical meaning, fostering belonging, and reframing audience relationships may reduce anxiety and increase flow (Ripani and Matei, in press) (see text footnote 1). However, it is important to note that our study did not employ validated measures of flow or anxiety, and the connections drawn here are interpretive, based on instructor accounts rather than direct psychological assessment.

The report of a student who started asking their studio teacher more questions is particularly significant in a context where disagreeing with a revered teacher can feel risky (Foletto, 2018). A participant reported stronger perceived score connection through hands-on structural reduction among students. Reduction might indeed balance fixed harmonic pillars with performer-determined parameters, avoiding both prescription and laissez-faire. Aligned with this, Cleland and Fleet (2021) advocate a musicianship-centered aural-skills pedagogy that integrates harmony, analysis, theory, improvisation, real-world and non-Western musics, and embodied approaches (e.g., Dalcroze).

Another reported benefit was identifying new ways to interpret scores and recognizing the indeterminacy of notation. Performers should be treated as interpreters, not transmitters, expanding room for negotiated meaning (Cook, 2013; Goehr, 1994; Leech-Wilkinson, 1984). In our data, courses 1 (P1), 5 (P5), and 9 (P9) engaged repertoire via HIP or explicit negotiation, often offering a vital second perspective beyond the principal teacher; multiple-teacher setups can foster critical evaluation, whereas single-teacher arrangements may encourage compliance (Gaunt, 2009).

#### Student reticence

Reluctance to improvise reflects internalized WCM ideology prioritizing flawless text reproduction and fuelling embarrassment and fear (Ayerst, 2021). Compared with jazz musicians, classical players emphasize tone/technique over memorization or improvisation and often report less pleasure (Benedek et al., 2014); training offers little improvisation despite its historical ubiquity and the shaky basis of “historical” prohibitions (Taruskin, 1995). The composer–performer split and industrial-era discipline helped recast improvisation as the opposite of composition, making today's precise text-reproduction with improvisation-avoidance a historical anomaly (Müller, 2023). Lowering the barrier means framing improvisation broadly—ornamentation, parameter variation (tempo, vibrato, articulation, dynamics, timbre), continuo-style chordal work, and thematic variation, i.e., recycling materials, as legitimate creative practice (Hill, 2017, 2018).

Some students' reticence regarding less conventional course content may reflect low tolerance of ambiguity (Yu et al., 2022) and lower openness to experience (Jack and Smillie, 2019). Conservatoire norms (master–apprentice pedagogy, score-obedience, and mistake aversion) discourage improvisation (Kingsbury, 1988; Gaunt, 2011; Carey and Grant, 2015; Bull, 2019; Scharff, 2018). Werktreue and text-centrism cast improvisation as illegitimate beside “great works” (Goehr, 1994; Taruskin, 1995; Cook, 2013), and students internalize these values and self-police (Leech-Wilkinson, 2020). Empirically, youth orchestras defended hierarchies (Bull, 2019), and older Sistema students resisted social-action aims, preferring European repertoire, consistent with norm internalization and effort-justification/loss-aversion dynamics (Baker, 2021). In teacher-worship contexts, a fixation on technique can push students into compliant performance at the expense of intrinsic musical motivation (Gaunt, 2009).

#### Employability: the good and the bad

Course content was viewed as potentially beneficial for widening students' horizons and employability, while also contentious, since less conforming ideas ran counter to established institutional (particularly orchestral) cultures.

Creative approaches can fit portfolio/protean careers in a competitive, non-linear industry where musicians juggle roles (Bartleet et al., 2019). Entrepreneurship here need not mean narrow venture logics: it includes opportunity recognition, resilience, career self-management, and, where needed, small-enterprise skills (Bridgstock, 2012). HME can scaffold this by building career identity, helping students spot values-aligned opportunities, and using experiential, community-of-practice projects, while broadening notions of success and making work–life trade-offs explicit (Bridgstock, 2012; Teague and Smith, 2015). Persistent dissatisfaction often reflects weak HME–industry links, scarce placements, tech gaps, limited business/legal literacy, thin networks, and narrow stylistic/cultural exposure, amid precarious funding, freelance instability, and rising AI pressures (Guile, 2006; Bartosova, 2011; Bennett, 2016; Bennett et al., 2015; UK Parliament, 2024).

Rather than reject entrepreneurship, HME can recast it as dynamic, socially embedded artistry or social/cultural entrepreneurship (Tullberg, 2025; Gaunt et al., 2021), and complement this with pedagogy that decenters one-way teaching, cultivates critical reflection, and foregrounds collaboration, preparing graduates to create work with communities, not only for markets (Tullberg, 2025).

Participants doubted near-term employability because the labor market rewards conformity. Auditions and orchestral work prize fidelity and blend over individuality; jurors expect players to “do justice to the score,” bounding creativity within style (McCormick, 2017). Orchestral culture is hierarchical and accuracy-driven with limited interpretive control, and selection systems leave little space for personal voice; a century of recordings and one-to-one pedagogy has further narrowed what juries expect, making novel readings sound anomalous (Dobson, 2010; Goldin and Rouse, 2000; Philip, 2004; Leech-Wilkinson, 2009, 2020). While some ensembles innovate formats or leadership, e.g., memorized performances (Aurora Orchestra, n.d.); conductor-free models (Orpheus Chamber Orchestra, n.d.), they rarely license score-level deviation; thus courses may build critical, creative agency, but payoffs are long-term and incremental within an industry that cherry-picks history to defend a homogeneous “modernist” style (Taruskin, 1995; Leech-Wilkinson, 2020; Bull, 2024).

### RQ4: instructors' perceptions of institutional context

#### Tension between the innovative courses and the institutional culture

Instructors described skepticism from colleagues and examination panels, the primacy of one-to-one studio instruction as the core business of conservatoires, funding pressures that pushed optional creative modules to intermittent scheduling, and assessment regimes that privilege accuracy over creative risk-taking.

One-to-one studio teaching is often seen as the privileged site of “real” learning, where planning/self-evaluation beyond repertoire choice is limited and trust is the engine of progress. While one-to-one tuition is variable (Carey and Grant, 2015) and often highly beneficial in healthy relationships (Matei and Ungureanu, 2026), critics note risks of dependency, poor transfer beyond the studio, and constrained autonomy (Gaunt, 2011). Complementary structures—workshops/studio seminars, team-teaching, and informal/peer learning—can offset such risks of the one-to-one model, though each has trade-offs (Gaunt, 2009; Carey and Grant, 2015). Visiting/part-time teachers can bring strong professional links (Bennett, 2009), underscoring the case for targeted teacher development. Institutionally, leaders juggle funding/legitimacy with innovation in a frictional culture defaulting to individualized one-to-one; decentralized authority and leadership turnover further complicate change (Ski-Berg et al., 2024).

Calling non-canonical work “too childish” can be seen as genre policing, not neutral taste: it protects classical music's status hierarchy by valorizing “seriousness,” score-fidelity, and correction-driven studios, while relegating playful, exploratory, or student-led work to the extracurricular, framings students then internalize (Bull, 2019; Bull and Scharff, 2017; Bull, 2024). These white middle-class norms code classical as “proper/deep” and other genres as “McDonald's music” (Bull, 2024). Playful pedagogy can counter this by legitimizing experimentation, risk-taking, and creative ideation, reducing mistake-aversion and resisting neoliberal performativity (Nørgård et al., 2017; Koeners and Francis, 2020).

Pedagogies vary widely (master–apprentice to co-learner; solo to team teaching) across contexts (Sætre et al., 2025), yet many HME teachers lack formal training or teaching qualifications (Norton et al., 2019; Boyle, 2020). Unsurprisingly, many teach as they were taught. A shift toward an improviser–composer–performer teacher identity is recommended (Campbell et al., 2016). Most studio faculty are part-time performers, limiting professional development uptake; institutions should tailor incentives and access (Duffy, 2016). Teachers often feel partly responsible for student wellbeing but underprepared, citing time/funding and limited professional development (Norton, 2016).

Score-fidelity priorities extend to leadership, where broadening pedagogy can be framed as “eroding standards” (Duffy, 2013). Terms like collaboration or interdisciplinarity can trigger defensiveness. Leaders report mixed signals: more openness among younger staff, but a hands-off stance that leaves group/collaborative teaching to individual discretion rather than embedding it (Rumiantsev et al., 2020). Students note competitive dynamics among senior teachers (Almqvist and Werner, 2023).

#### A manifesto in disguise: shielding innovation for change

Instructors often eased resistance by historicizing new practices, an expedient way to align with entrenched score-fidelity and genre policing. Innovations were vulnerable when seen to threaten the core business of one-to-one repertoire/technique training (Duffy, 2016). Change is politically hard in conservatoires (Parncutt, 2007); effective tactics include building on existing strengths, aligning with studio teachers' goals, addressing administrators' metrics, and evidencing outcomes. Bringing faculty and leadership together to embed goals in mission statements can legitimize direction, though formal adoption does not guarantee implementation.

Research-informed, highly creative approaches are often too new to be fully embedded in HME, and studio teachers' caution makes a research–pedagogy lag unsurprising. Its length hinges on how researchers engage with practitioners' critiques and adapt. Parncutt (2007) counters fears of undermining teacher–student bonds: great performers often had multiple teachers; students have a right to diverse sources; and new proposals are anti-positivist: contestable, not dogma. Students can be trained to evaluate claims independently to ease adoption without threatening studio relationships.

Participants recognized that change is slow. Even when they knew their content would create tensions with other influences, they felt responsible for offering students an alternative perspective. Tellingly, improvisation was often kept at the margins—useful, but carefully cordoned off so it would not “interfere” with repertoire. This helps explain why we found few courses that explicitly set out to disrupt prevailing approaches to repertoire.

#### Facilitating change through institutional forces

Change takes root fastest in institutions that are open, research-oriented, interdisciplinary, flexible in assessment, and backed by supportive leadership. Cross-sector models, where performer training sits inside universities, blur practice/scholarship boundaries and enable more experimental curricula than performance-only conservatoires (Ford, 2010). Policy levers also matter: national initiatives and accreditation standards that embed improvisation/creativity legitimize change. Internally, three moves help: targeted teacher development (improviser-composer-performer identities), curricular oversight that reviews relevance and evidence of impact, and co-design forums (faculty–student–admin–external “think tanks”), supported by consortia/conferences to share models (Campbell et al., 2016; Kertz-Welzel, 2024).

Creativity underpins professional readiness and motivation; students equate it with interpretive freedom, not mechanical repetition (Dushniy et al., 2025). Assessment should therefore extend beyond accuracy and canonical fidelity to recognize multiple virtuosities (process skills, ensemble creativity, originality, facilitation) and diverse outcomes (MacDonald and Saarikallio, 2024). Practically, this means weighting processual artifacts (portfolios, rehearsals, reflective notes), valuing interpretation, improvisation, and collaboration as legitimate criteria (Dushniy et al., 2025), and favoring formative, autonomy-supportive assessment (e.g., pass/fail or banded schemes) over fine-grained ranking (Saltari and Kokkidou, 2024). Institutions should co-design criteria with staff and students to align purpose and practice and keep a live dialogue on what “creative performance” means and how it is judged (Creech et al., 2013).

#### Strengths and limitations

This study advances understanding of norm-challenging pedagogy in Western classical music higher education in four ways. First, it offers rich, contextualized accounts of how critically oriented instructors design and deliver courses that expand interpretive autonomy, providing practical examples that may inform curricular development elsewhere. Second, our framework, distinguishing repertoire-based from non-repertoire designs, and historically informed from ahistorical deviation, maps approaches along a continuum of norm challenge. Third, documenting insider strategies (e.g., historical framing) highlights the pragmatic navigation required to innovate within resistant institutions. Fourth, by bridging psychology and musicology, the study models the kind of multidisciplinary collaboration needed to address the intertwined dynamics of pedagogy, practice, history, and wellbeing in conservatoire contexts.

Several limitations warrant attention. First, our purposive sampling and recruitment likely attracted instructors already sympathetic to the practices examined. Second, circularity between our theoretical framework, sampling, and analysis means the findings describe how a particular group of instructors work, not how common these approaches are across HME. Third, the sample is concentrated in Europe and the UK, and anonymised national contexts limit generalizability and preclude geographic comparison within the dataset. Ethical anonymisation also limits some contextual interpretation. Fourth, provision varied widely (from single modules to full degree programmes), complicating cross-course comparison; a cleaner split between repertoire-focused and skills-based courses might increase clarity but would risk obscuring the hybrid nature of much experimental pedagogy. Fifth, reported outcomes related to wellbeing and employability are based on instructor perceptions. While valuable for understanding how instructors conceptualize effects, they cannot establish causal relationships or student-level outcomes. Observations may be subject to confirmation bias and limited visibility. Importantly, psychological constructs such as performance anxiety, flow, and related wellbeing accounts were not directly measured using validated instruments in this study; rather, they are reported only via instructors' accounts of their observations and interpretations of student experiences. Sixth, English-only interviews introduced a linguistic and cultural filter: participants from non-Anglophone contexts may have expressed ideas differently in their native languages, and those with limited English proficiency were excluded. This limitation should be considered when interpreting nuances in participant accounts.

#### Directions for future research

Several future research directions follow. First, studies should directly assess student experiences using validated measures of performance anxiety, flow, autonomy, and wellbeing to move beyond instructor perceptions and document student-level outcomes. Second, longitudinal designs tracking students through and beyond training could examine how exposure to norm-challenging pedagogy relates to later professional practice and career trajectories. Third, audience research remains underexplored: if the norms challenged in these courses also shape listener experience, examining audience responses to performances informed by norm-challenging pedagogy could strengthen the case for curricular change. In particular, research should test how listeners respond to interpretive diversity, historical deviation, and improvisatory freedom in classical performance contexts.

Comparative research across national and institutional contexts could clarify how cultural and structural factors shape opportunities for pedagogical innovation. Work from outside Europe and the UK is particularly needed to examine whether and how norm-challenging pedagogy operates in other classical training traditions, and what institutional dynamics enable or constrain innovation. Research on assessment practices, and their effects on student risk-taking, could inform strategies for creating space for experimentation without career penalty; the assessment flexibility identified here suggests procedural change may be a tractable lever for wider reform. Finally, implementation-focused research—case studies of successful institutional change, identification of enabling conditions, and adaptable pedagogical resources—could support instructors seeking to introduce norm-challenging elements into their teaching.

## Conclusion

The College Music Society's manifesto argues that twenty-first-century musicians should improvise, compose, perform, teach, and think critically, supported by curricula that enable multiple identities and embodied, cross-arts practice (Campbell et al., 2016). MacDonald and Saarikallio (2024) similarly call for new virtuosities—creative, social, and technical. In our data, instructors integrated theory/aural skills with application and treated performance, improvisation, and composition as core rather than elective, noting that improvisation can reduce accuracy pressure, fear of mistakes, and performance anxiety (Duffy, 2016). Cross-disciplinary, collaborative pedagogy also challenges the master–apprentice norm and supports broader, healthier musical identities (Rumiantsev et al., 2020; MacDonald and Saarikallio, 2024).

We advocate a dialogical approach to tradition, which can reduce fears of “denying” the past: following Gadamer, tradition is continually handed over through interpretation, and works remain unfinished, constantly remade (Smith and Peters, 2024). The future lies neither in an “idyllic past” nor in uncritical technophilia, but in building on inherited practices while responding to present needs. In this regard, we advocate giving classical musicians greater permission to take risks and pursue unconventional approaches, supporting sustainability, performer fulfillment, and cultivating wider audiences. Audience-impact research could further shift attention from “dead composers” to living listeners and communities; a global manifesto likewise frames norm-challenging as integral to wellbeing (Matei et al., in review).^2^ This is not innovation-as-neoliberal productivity, nor a claim that musicking sits outside ideology (Osborne, 2003). Rather, following Green (2003), interrogating ideology clarifies which values are privileged and with what effects, without collapsing into relativism. Freedom involves moving between identifications (Ayerst, 2021; after Balibar, 2015), and ideologies can be evaluated, with some better than others. Because education regulates practice and opportunity, creative approaches should be treated as equal options, not marginal “labs” (Leech-Wilkinson, 2020). Without explicit debate, critical thinking stalls: boundaries matter, but where we draw them, and why, must be argued.

We acknowledge music's subjectivity and treat non-historical rationales and atypical approaches as equally legitimate, acknowledging that paradigmatic change can reimagine rather than dilute excellence and rigor. There is greater risk in refusing ideological scrutiny and thereby foreclosing the possibility of better, more relevant norms. As Campbell et al.'s manifesto argues,

“if Bach, Beethoven, Mozart, Schumann, and Liszt were alive today, their musical lives would more likely resemble today's creative jazz artists (and other improvisers-composers/performers) than the interpretive performance specialists whose repertory was created in and for another time and place” (Campbell et al., 2016, pp. 1–2).

## Data Availability

The original contributions presented in the study are included in the article/supplementary material, further inquiries can be directed to the corresponding author.

## Footnotes

1 Ripani, G., and Matei, R. (in press). Risk and protective factors of music performance anxiety and flow: a scoping review. *Psychol. Music*.

2 Matei, R., Ackermann, B., Allen, J., Alpagut, U., Ananda-Owense, K., Araújo, L., et al. (in review). Health promotion in tertiary music institutions: a position paper. *Arts Educ. Policy Rev*.
